# 经皮冷冻治疗644例肺癌的常见并发症分析及处理

**DOI:** 10.3779/j.issn.1009-3419.2010.08.16

**Published:** 2010-08-20

**Authors:** 立志 牛, 静 王, 亮 周, 炳辉 吴, 峰 穆, 海波 李, 勇 胡, 以则 胡, 建生 左, 克成 徐

**Affiliations:** 1 510305 广州，中国科学院广州生物院附属复大医院肿瘤科 Department of Medical Oncology, GIHB Afliated to Fuda Hospital, Chinese Academy of Sciences, Guangzhou 510305, China; 2 510305 广州，广州复大肿瘤医院肿瘤科 Department of Medical Oncology, Fuda Cancer Hospital, Guangzhou 510305, China

**Keywords:** 冷消融, 冷冻治疗, 肺肿瘤, Cryoablation, Cryotherapy, Lung neoplasms

## Abstract

**背景与目的:**

肺癌患者大部分确诊时已属中晚期，失去了手术机会。冷冻疗法是一种可选择的安全有效的局部消融疗法。本研究旨在分析经皮冷冻治疗中晚期肺癌的常见并发症。

**方法:**

644例不能手术切除的肺癌患者在CT和/或B超下行经皮冷冻治疗，观察心血管系统和呼吸系统并发症的发生率，并采取相应的处理措施。

**结果:**

肺癌经皮冷冻治疗的一般并发症程度较轻，对症处理即可恢复。严重并发症包括心脏骤停、血气胸等，重在预防。

**结论:**

经皮冷冻治疗不可切除的肺肿瘤是一种简单、有效且相对安全的方法，但要注意预防并发症的发生。

## Abstract (en)

**Background and objective:**

Most lung cancer patients present an advanced stage at diagnosis without suitable surgical chances. Cryotherapy has been applied to the advanced lung cancer with safety and effectiveness. The aim of this study is to analyze the most common complications that occur after percutaneous cryoablation when applied to advanced lung cancer.

**Methods:**

Total 644 lung cancer patients had been treated with percutaneous cryoablation guided by ultrasound and/or CT scan. The cardivascular and respiratory complications were monitored.

**Results:**

Complications were relatively minor and could be controlled with routine methods. Serious complications included cardiac arrest and hemopneumothorax, and thus preventative steps should be taken.

**Conclusion:**

Percutaneous cryoablation for lung cancer is a simple, effective and relatively safe method, but attention should be paid to the possible complications during cryoablation.

肺癌是全世界癌症死因的重要原因^[1, 2]^，也是我国发病率和病死率最高的癌症之一。本病多在40岁以上发病，好发于老年人，大部分得以确诊时已属中晚期，失去了手术机会。经皮冷冻可用于治疗不能手术切除的中晚期肺癌，且局部肿瘤控制率满意^[3-5]^。复大医院肿瘤科自2001年8月-2008年8月采用经皮冷冻治疗644例不能手术切除的原发性肺肿瘤，疗效满意，结果证明经皮冷冻治疗是一种有效、安全、微创的疗法，现将其冷冻治疗过程中出现的最常见的心血管系统和呼吸系统的并发症报告如下：

## 1 材料与方法

### 1.1 临床资料

本组644例肺癌患者，其中男性467例，女性177例；年龄为14岁-95岁，中位年龄为65岁；其中小细胞肺癌（small cell lung cancer, SCLC）42例，非小细胞肺癌（non-small cell lung cancer, NSCLC）602例；在NSCLC中，其中鳞癌268例，腺癌234例，未分化癌69例，肺泡细胞癌31例。本组病例中，肺部肿瘤直径在1 cm-25 cm之间，平均6.8 cm。

### 1.2 治疗方法

使用Cryocare Surgical System（CRYO-20型）冷冻外科系统。早期在CT和/或B超引导下推进带芯穿刺针，通过导丝经皮引入鞘管套后抽出内芯，将氩氦刀顺着穿刺鞘插入肿瘤固定，后期直接用直径为2 mm-1.7 mm的冷冻探针直接穿刺。根据瘤体大小、形状、部位等可选择同时插入2支-4支冷冻探针。如果肿瘤 < 2 cm时，插入1支直径为2 mm的探针即可；如果肿瘤在2 cm-4 cm之间，则插入2支冷冻探针；如果肿瘤≥5 cm时，则可插入3支-4支探针。CT扫描和/或B超证实刀头位置准确后，开启氩氦刀冷冻系统，开始输入氩气，冷冻温度显示-130 ℃至-140 ℃，持续冷冻15 min后，然后停输氩气，改输氦气复温5 min，使温度显示为10 ℃至20 ℃，为1个冷冻-复温循环。此时复查CT或B超，观察冰球范围，冰球未覆盖肿瘤时要“补刀”。然后再重复1次冷冻-复温循环，待2次循环结束后复查CT或B超。本组病例均在局麻下经CT或B超引导穿刺行经皮冷冻治疗。

## 2 结果

主要有心血管系统和呼吸系统的并发症。

心血管系统的并发症主要有：①心动过缓（即心率 < 60次/分），79例，占本组病例的12.3%，使用阿托品、多巴胺等药物对症处理后恢复；②室性早搏，20例，占本组病例的3.1%，使用利多卡因、可达龙等药物处理后恢复；③房颤，2例，占本组病例的0.3%，使用西地兰对症处理后恢复；④心脏骤停，1例，占本组病例的0.2%，采用心脏按压、气管插管、电击除颤等抢救措施后恢复；⑤低血压，95例，占本组病例的14.8%，使用多巴胺、肾上腺素等药物处理后恢复。以上并发症的病例，无一例死亡（表 1）。

**1:** 心血管系统并发症 The major complications of cardiovascular system

Complications	Cases (*n*)	Percent (%)	Deaths (*n*)
Bradycardia (heart rate < 60 per minute)	79	12.3	0
Vntricular premature beat (VPB)	20	3.1	0
Atrial fibrillation	2	0.3	0
Cardiac arrest	1	0.2	0
Hypotension	95	14.8	0

呼吸系统的并发症主要有：①哮喘发作，9例，占本组病例的1.4%，使用糖皮质激素、氨茶碱等药物解痉平喘，重者用呼吸机辅助呼吸等对症处理；②咯血，357例，其中大咯血4例，分别占本组病例的55.4%和0.6%，可使用止血剂对症处理；③血气胸，209例，占本组病例的32.5%，可采用胸穿、闭式引流等方法对症处理；④肺部感染，43例，其中4例发展为呼吸衰竭，分别占本组病例的6.7%和0.6%，可使用抗生素对症处理，重症呼吸衰竭患者可行气管切开、呼吸机辅助呼吸；⑤发热，105例，占本组病例的16.3%，使用药物对症处理。以上呼吸系统并发症的病例，经对症处理、积极抢救后，血气胸死亡1例，呼吸衰竭死亡2例。本组所有病例并发症相关总死亡率为0.47%（表 2）。

**2:** 呼吸系统并发症 The major complications of respiratory system

Complications	Cases(*n*)	Percent (%)	Deaths (*n*)
Asthma attack	9	1.4	0
Hemoptysis (massive hemoptysis)	375⑷	55.4 (0.6)	0
Pneumohemothorax	209	32.5	1
Lung infection (respiratoryfailure)	43⑷	6.7 (0.6)	2
Fever	105	16.3	0

## 3 讨论

经皮肺冷冻的并发症主要有咯血、气胸、血胸、胸腔渗液、并发感染等。王洪武等^[6]^报道46.8%的患者在冷冻治疗后发生咯血，16.1%有发热，25.8%发生气胸，19.4%有胸腔积液。Kawamura等^[3]^治疗的22例经皮冷冻治疗中有11例（50%）并发气胸，7例（27%）有小量胸腔积液，9例（41%）咯血，上述三种不良反应在保守治疗后均消失；1例（4.5%）并发膈神经麻痹，系因肿瘤位于左膈神经旁、冷冻时神经受累所致。与以上文献报道相比，本组病例出现严重并发症比率相对较低，冷冻治疗总体上是安全有效的，术中无一例死亡，仅有3例死于并发症。可见，防治冷冻后并发症至关重要。下面对该组病例出现的并发症的发生原因及处理方法做一简要分析：

心动过缓、低血压：冷冻降温对心血管的影响表现为心率减慢、心肌收缩力降低，深低温甚至可引起心脏停搏，本组患者有79例出现心动过缓，血压降低，收缩压 < 90 mmHg。一般给予阿托品、多巴胺等药物、全身保温等处理后均可恢复。

室性早搏、房颤：肺癌多为老年患者，大多合并心脏病等因素，冷冻过程中加之心理紧张所致心率加快、心律不齐，出现室性早搏甚至房颤。所以在术前医生应做好预防措施，控制原发病。一旦发生此类并发症，室性早搏采用利多卡因、可达龙等调节心律，房颤采用西地兰减慢心率等对症治疗，患者病情可好转。

心脏骤停：由于温度快速下降，当低温血液从四肢末端流回心脏，可导致心脏病发作甚至心脏骤停，是冷冻过程中最严重的并发症之一。本组病例中有1例出现心脏骤停，可能与肿瘤较大贴近并挤压心脏、冰球较大、冷冻时间较长等有关。该病例经积极抢救后病情平稳。所以在冷冻过程中严密监测患者生命体征和及时处理并发症非常重要，同时手术室要准备气管插管及除颤仪等抢救设备，重在预防。

哮喘发作：较少见，但却是严重并发症，哮喘发作常与慢性肺病史、冷冻刺激及肿瘤免疫抗原性改变有关。所以手术前要处理和控制患者的原发病，提前给予激素等预防用药，并做好预防措施。一旦发生此并发症，需要紧急处理。轻症需用激素、氨茶碱等舒张气管，解痉平喘；重症者立即气管插管或气管切开，呼吸机辅助呼吸。此外，哮喘发作还与术后肿瘤组织坏死后容易继发感染有关，加强抗生素治疗是十分必要的。本组病例经对症处理后，症状均缓解。

咯血：主要与穿刺及冷冻后肿瘤坏死有关，本组病例出现4例大咯血，其中2例为癌性空洞，可能是术后咳血的一个主要影响因素。对大咯血患者应及时采用止血剂、垂体后叶素静脉滴注，有空洞的患者要加强抗菌治疗预防感染。本组病例经积极对症治疗后均恢复。

血气胸：是胸腔穿刺常见的并发症，本组病例发生率为32.5%，故在穿刺时要避免穿刺肺大泡及肺气肿的肺组织，以免张力性气胸的发生。出现血气胸并发症的患者需行胸穿闭式引流处理，本组有1例经治疗无效死亡。

肺部感染（呼吸衰竭）：术后有肺部感染病例，严重者可能发展为呼吸衰竭，处理此并发症时要积极抗感染处理，严重者行气管插管或气管切开、呼吸机对症支持等。本组有2例因呼吸衰竭而死亡。

发热：冷冻后术区肿瘤细胞缺血坏死，产生内生致热原（endogenous pyrogen, EP），机体吸收坏死物引起全身性应激反应，有利于清除病原体。如果患者体温低于38.5 ℃时，一般不需要特殊处理，患者体温多可自行恢复正常。若体温过高，且持续时间过长，则可能为伤口感染或肿瘤本身大范围坏死所致，此时要严密监测患者生命体征，采用抗感染等药物对症处理。

总之，经皮冷冻是一种重要的消融技术，可用于治疗无法手术切除的中晚期肺癌，近期存活率高，疗效满意。目前冷冻治疗已成为经多种治疗方法无效或已失去手术治疗时机的晚期癌症患者的首要选择，但同时其亦难以避免并发症的发生。所以在行冷冻治疗时，术前、术中和术后均要预防各类并发症的发生，一旦并发症无可避免，我们要积极采取急救措施，最大限度降低并发症对患者机体的伤害，提高生存质量。
